# A Fast Framework for Abrupt Change Detection Based on Binary Search Trees and Kolmogorov Statistic

**DOI:** 10.1155/2016/8343187

**Published:** 2016-06-16

**Authors:** Jin-Peng Qi, Jie Qi, Qing Zhang

**Affiliations:** ^1^College of Information Science & Technology, Donghua University, Shanghai 201620, China; ^2^Australia e-Health Research Centre, Csiro Computation Informatics, Brisbane, QLD 4060, Australia

## Abstract

Change-Point (CP) detection has attracted considerable attention in the fields of data mining and statistics; it is very meaningful to discuss how to quickly and efficiently detect abrupt change from large-scale bioelectric signals. Currently, most of the existing methods, like Kolmogorov-Smirnov (KS) statistic and so forth, are time-consuming, especially for large-scale datasets. In this paper, we propose a fast framework for abrupt change detection based on binary search trees (BSTs) and a modified KS statistic, named BSTKS (binary search trees and Kolmogorov statistic). In this method, first, two binary search trees, termed as BSTcA and BSTcD, are constructed by multilevel Haar Wavelet Transform (HWT); second, three search criteria are introduced in terms of the statistic and variance fluctuations in the diagnosed time series; last, an optimal search path is detected from the root to leaf nodes of two BSTs. The studies on both the synthetic time series samples and the real electroencephalograph (EEG) recordings indicate that the proposed BSTKS can detect abrupt change more quickly and efficiently than KS, *t*-statistic (*t*), and Singular-Spectrum Analyses (SSA) methods, with the shortest computation time, the highest hit rate, the smallest error, and the highest accuracy out of four methods. This study suggests that the proposed BSTKS is very helpful for useful information inspection on all kinds of bioelectric time series signals.

## 1. Introduction

Abrupt change detection is to identify abrupt changes in the statistical properties of a signal series, which occur at unknown instants [1–3]. These changes are interesting because they are indicative of qualitative transitions in the data generation mechanism (DGM) underlying the signals. Currently, CP detection has attracted considerable attention in the fields of data mining and statistics, and it has been widely studied in many real-world problems, such as atmospheric and financial analyses [1], fault detection in engineering system [4, 5], climate change detection [6], genetic time series analyses [7], signal segmentation [8, 9], and intrusion detection in computer network [4].

In community of statistics, some nonparametric approaches for CP detection have been widely explored. For example, KS statistic quantifies a distance between the empirical distribution function of the sample and the cumulative distribution function of the reference distribution or between the empirical distribution function of two samples [10, 11]. Also, KS statistic and its modified versions are broadly investigated on many application fields, for example, testing hypotheses regarding activation in blood oxygenation level-dependent functional MRI data [12], modeling the cumulative distribution function of rub-induced AE signals, quantifying the goodness of fit to offer a suitable signal feature for diagnosis [13], as well as abrupt change detecting on EEG signals [14], and gene expression time series [15]. Meanwhile, as for the model-related statistic approaches, some modified cumulative sum (CUSUM) methods provide the asymptotic distributions of test statistics and the consistency of procedures and behave better in finite samples and have a higher stability with respect to the time of change than ordinary CUSUM procedures [16]. The CUSUM method and its revised versions have been widely applied to detect the structural breaks in the parameters of stochastic models, as well as the abrupt changes in the regression parameters of multiple time series regression models, such as multiple CP detection in biological sequences [17], abrupt change detection in the regression parameters of a set of capital asset pricing data related to the Fama-French extension of the CAPM [16], and abrupt change detection in a shape-restricted regression model [18].

On the other hand, SSA is a powerful technique for time series analyses. SSA is nonparametric and requires no prior knowledge on the properties of time series signal [19]. The main idea of SSA is applied in the principal component analyses on the trajectory matrix with subsequent reconstruction of the original time series. SSA has been proved to be very successful and has already become a standard tool in the analyses of climatic [10], meteorological, and geophysical time series [11, 19]. Currently, SSA has been successfully applied in the real time series recordings, for example, abrupt change analyses on EMG-onset detection [12] and CP detection in time series [13]. Although SSA is a model-free method, it is not scalable to large-scale datasets, because it is time-consuming and sometimes invalid for time series analyses with less significant data fluctuation.

In addition, Wavelet Transform (WT) is another important tool for time series analyses [14, 15, 20–23]. WT has been widely applied in anomaly detection, time series prediction, image processing, and noise reduction [15, 23–25]. WT can represent general function at different scales and positions in a versatile and sophisticated manner, so that the data distribution features can be easily extracted from different time or space scales [25, 26]. As a simple WT, Haar Wavelet (HW) owns some attractive features including fast implementation and ability to analyze the local features. HW is very useful to find abrupt changes of discontinuity and high frequency in time series, so it is a potential candidate in modern electrical and computer engineering applications, such as signal and image compression, eye detection [27], abnormality detection on time series [28, 29], and abrupt change detection on autoregressive conditional heteroscedastic processes [30].

However, all of these methods above are time-consuming and sometime invalid for abrupt change detection near the left or the right boundary, especially for insignificant data fluctuation in large-scale time series. To resolve these problems, we propose a fast framework for CP detection based on binary search trees and a modified KS statistic, termed BSTKS for short. In this novel method, first, two BSTs are derived from a diagnosed time series. Second, three search criteria are introduced in terms of the statistic and variance fluctuations between two adjacent time series segments, and then an optimal search path is detected from the root to leaf nodes of two BSTs. Last, the proposed BSTKS and other KS, *t*, and SSA methods are tested on both the synthetic time series and real EEG recordings and evaluated in terms of computation time, hit rate, error, accuracy, and area under curve (AUC) of Receiver Operating Characteristic (ROC) curve analyses.

In general, for a certain bioelectric signal, an abrupt change means an important transition of biological functions or health states before and after a strong attack or an acute perturbation from internal or external environment. Therefore, it is very necessary to not only discern abrupt change from all kinds of physiological and psychological time series signals, but also inspect the significant fluctuation between adjacent time series segments with different scales. The following sections focused on not only presenting the framework of the proposed BSTKS method through theoretical foundation, simulation, and evaluation, but also discussing how it can more quickly and efficiently detect abrupt change on both synthetic and real bioelectric EEG signals than other existing KS, *t*, and SSA methods. The rest of this paper is organized as follows.  Section 2 gives the preliminary of abrupt change by introducing the statistic and variance fluctuations between two adjacent time series segments.  Section 3 implements the integrated framework of the BSTKS method in terms of three search criteria in detail.  Section 4 provides some representative experiments by using the synthetic time series and real EEG recordings and then analyzes the performance of BSTKS by comparing with other KS, *t*, and SSA methods.  Section 5 gives summary and conclusion from previous sections.

## 2. Preliminary

### 2.1. Statistic Fluctuation

KS statistic is sensitive to differences in both location and shape of the cumulative distribution functions (c.d.f) of two samples. The null distribution of KS statistic is calculated under the null hypothesis that the two samples are drawn from the same distribution or one sample is drawn from the reference distribution. To detect an abrupt change from a diagnosed time series *Z*, we define the statistic fluctuation between two adjacent segments within *Z* by means of KS statistic as follows [1, 4, 19].

**Definition 1 .**

Supposing a time series sample, *Z* = {*z*_1_,…, *z*_*N*_}, one observes

(1) $$\begin{array}{c} Z=f\left(\;\frac{i}{n}\;\right)+X,\;i=1,\ldots ,N, \end{array}$$

where *X* = {*x*_*i*_}_*i*=1,…,*N*_ is a set of the discrete and centred i.i.d random variables and *f* is a noisy mean signal with unknown distribution. The statistic fluctuation between two adjacent segments *Z*_*L*_ = {*z*_*a*_,…, *z*_*c*_} and *Z*_*R*_ = {*z*_*c*+1_,…, *z*_*b*_} is defined as

(2) $$\begin{array}{c} S_{mn}\left(\;x\;\right)={\left(\;\frac{mn}{m+n}\;\right)}^{1/2}\underset{x\in R}{\sup}\left|\;F_{m}\left(\;x\;\right)-G_{n}\left(\;x\;\right)\;\right|, \end{array}$$

in which *F*_*m*_(*x*) and *G*_*n*_(*x*) are the c.d.f of *Z*_*L*_ and *Z*_*R*_, respectively; *m* = *c* − *a*, *n* = *b* − *c* − 1, and *m* + *n* ≤ *N*. Supposing the hypothesized *F*_*m*_(*x*) and *G*_*n*_(*x*) in (2) are not available, we can derive the empirical cumulative distribution functions (e.c.d.f) of *F*_*m*_(*x*) and *G*_*n*_(*x*) from *Z*_*L*_ and *Z*_*R*_. Then, *F*_*m*_(*x*) and *G*_*n*_(*x*) can be redefined as

(3) Fmx=PmZL≤x=1m∑i=acIzi≤x,Gnx=PnZR≤x=1n∑j=c+1NIzj≤x,

where *F*_*m*_(*x*) and *G*_*n*_(*x*) count the proportion of the sample points below level *x*.

**Hypothesis 1 .**

In order to discern an abrupt change on *Z* in terms of statistic fluctuation defined above, we introduce KS test for two adjacent segments *Z*_*L*_ and *Z*_*R*_ in *Z* as

$\left(H_{0}\right)$ if *S*_*mn*_(*z*_*c*_) ≤ *δ*, no abrupt change occurs in *Z*;
$\left(H_{1}\right)$ if *S*_*mn*_(*z*_*c*_) > *δ*, abrupt change occurs in *Z*,

in which *δ* ∈ *R* is a threshold of the statistic fluctuation within *Z* belonging to an identical distribution. Then, we test (*H*_0_) against (*H*_1_) from observations. If an abrupt change *c* occurs in *Z*, there exists a value *c* satisfying *S*_*mn*_(*z*_*c*_) > *δ*, *z*_*c*_ ∈ [*z*_1_, *z*_*N*_], and *δ* ∈ *R*. In this hypothesis, we assume that the number, the location, and the size of the function *f* in (1) are unknown, and the upper bound of the statistic fluctuation *δ* is supposed to be known.

### 2.2. Variance Fluctuation

Provided the statistic fluctuation defined in (2) is insignificant enough, it is difficult to detect abrupt change near the left or the right boundary within *Z*, especially when sample size *N* gets smaller. Therefore, we need to introduce another variable to calculate the variance fluctuation between two adjacent parts within a time series sample.

**Definition 2 .**

Supposing two adjacent segments *Z*_*L*_ = {*z*_*a*_,…, *z*_*c*_} and *Z*_*R*_ = {*z*_*c*+1_,…, *z*_*b*_} in *Z* = {*z*_1_,…, *z*_*N*_}, the variance fluctuation between *Z*_*L*_ and *Z*_*R*_ is defined as

(4) $$\begin{array}{c} D_{mn}\left(\;c\;\right)=\underset{1\leq L,R\leq N}{sup}\left|\;\frac{1}{m}\overset{c}{\underset{L=a}{\sum }}z_{L}-\frac{1}{n}\overset{b}{\underset{R=c+1}{\sum }}z_{R}\;\right|, \end{array}$$

in which *m* = *c* − *a*, *n* = *b* − *c* − 1, and *m* + *n* ≤ *N*.

**Hypothesis 2 .**

(*H*_0_) If *D*_*mn*_(*c*) ≤ *β*, no abrupt change occurs at *c* in *Z*; (*H*_1_) if *D*_*mn*_(*c*) > *β*, abrupt change occurs at *c* in *Z*.

Here, *β* ∈ *R* is a variance threshold of time series *Z* which obeys an identical distribution. If there exists a value *c* satisfying *D*_*mn*_(*c*) > *β*, *z*_*c*_ ∈ [*z*_1_, *z*_*N*_], then an abrupt change occurs at *c* in *Z*.

## 3. Method

### 3.1. Two BSTs' Construction

**Figure 1:**
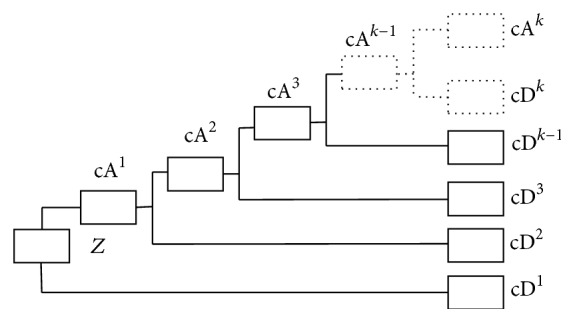
The diagram of a discrete time series *Z* decomposition by *k*-level HWT, which is composed of *k*-level cA and cD, that is, the average and difference coefficient vectors.

In the first part of the proposed BSTKS method, two BSTs, that is, BSTcA and BSTcD, are constructed from a time series sample *Z*, by using multilevel HWT. Generally, as shown in Figure 1, a discrete time series signal *Z* = {*z*_1_, *z*_2_,…, *z*_*N*_} can be decomposed into the *k*th-level trend cA^*k*^ and *k*-level fluctuations, that is, cD^1^, cD^2^,…, cD^*k*^, *k* = 1,2,…, log_2_ *N*. The *k*-level HWT is the mapping *H*_*k*_ defined as [13]

(5) $$\begin{array}{c} Z\overset{H_{k}}{\to }\left(\;cA^{k}\;|\;cD^{k}\;|\;cD^{k-1}\;|\;\cdots \;|\;cD^{2}\;|\;cD^{1}\;\right), \end{array}$$

and then, the mapping *H*_*k*_ can be represented by the approximation and detail coefficient matrices, termed McA and McD as follows:

(6) McA=cA1,1⋯cA1,N⋮cAk,j⋮cAM,100,McD=cD1,1⋯cD1,N⋮cDk,j⋮cDM,100,

where 0 ≤ *k* ≤ *M* = log_2_ *N* and 1 ≤ *j* ≤ *N*/2^*k*^.

Supposing the size of a diagnosed *Z* is divisible *k* times by 2, the *j*th element cA_*k*,*j*_ in cA^*k*^ and the *j*th element cD_*k*,*j*_ in cD^*k*^ can be denoted as

(7) cAk,j=12 ∧k∑i=abzi,cDk,j=12 ∧k∑L=aczL−∑R=c+1bzR,

where 1 ≤ *k* ≤ log_2_ *N* and 2^*k*^(*j* − 1) + 1 ≤ *i* ≤ *j∗*2^*k*^; *a* = 2^*k*^(*j* − 1) + 1, *c* = 2^*k*^(*j* − 1) + 2^(*k*−1)^, and *b* = 2^*k*^ *∗j*.

**Figure 2:**
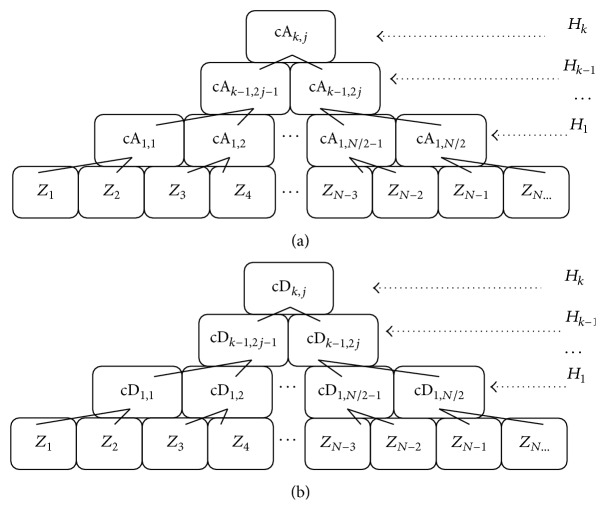
The diagrams of two binary trees, BSTcA and BSTcD, which are constructed by McA and McD, as well as the original time series *Z*.

During two BSTs' construction, as shown in Figure 2, the non-leaf nodes in BSTcA and BSTcD are assembled by the *k*-level coefficient vectors of McA and McD, respectively; and then the leaf nodes are derived directly from the original time series *Z*. Therefore, the features of abrupt change in *Z* can be reflected and distributed into the different non-leaf nodes of BSTcA and BSTcD, in accordance with the *k* level coefficient vectors in McA and McD.

### 3.2. CP Detection Based on Three Search Criteria

To find an optimal path towards the potential CP within a given time series *Z* quickly and efficiently, some search criteria need to be introduced, and then the data exceptions can be detected from the root to leaf nodes of two BSTs. As for the statistic fluctuation within BSTcA, first, a new variable *z*_*k*,*j*_ is defined according to a current non-leaf node cA_*k*,*j*_ in BSTcA,

(8) $$\begin{array}{c} z_{k,j}=\frac{1}{2^{k}}\left(\;\overset{b}{\underset{i=a}{\sum }}z_{i}\;\right)=\frac{\left(\sqrt{2}\right)\overset{\wedge }{\;}k}{2^{k}}cA_{k,j}, \end{array}$$

where 1 ≤ *k* ≤ log_2_ *N*, 1 ≤ *j* ≤ *N*/2^*k*^; *a* = 2^*k*^(*j* − 1) + 1, *b* = 2^*k*^ *∗j*, and *a* ≤ *i* ≤ *b*. Then, the statistic fluctuation between two adjacent segments *Z*_*L*_ = {*z*_*a*_,…, *z*_*c*_} and *Z*_*R*_ = {*z*_*c*+1_,…, *z*_*b*_} can be defined by a modified KS statistic as

(9) Smn′k,j=nmn+m1/2·1n∑iL=acIziL≤zk,j−1m∑iR=c+1bIziR≤zk,j,

in which *z*_*k*,*j*_ is a new element defined in (8); *m* and *n* stand for the sizes of *Z*_*L*_ and *Z*_*R*_, respectively; 1 ≤ *k* ≤ log_2_ *N*, 1 ≤ *j* ≤ *N*/2^*k*^; *a* = 2^*k*^(*i* − 1) + 1, *b* = 2^*k*^ *j*, and *c* = 2^*k*^(*j* − 1) + 2^(*k*−1)^. *S*_*mn*_′(*k*, *j*) measures the e.c.d.f difference between *Z*_*L*_ and *Z*_*R*_, and the larger *S*_*mn*_′(*k*, *j*) means the more significant statistic fluctuation between *Z*_*L*_ and *Z*_*R*_. Therefore, a potential abrupt change might occur at *c* in *Z* with more probability.

**Definition 3 .**

For a current non-leaf node cA_*k*,*j*_ in BSTcA, with its left and right-child nodes cA_*k*−1,2*j*−1_ and cA_*k*−1,2*j*_, the distance of e.c.d.f, *S*_*k*,*j*;*L*_, and *S*_*k*,*j*;*R*_ can be defined as

(10) Sk,j;L=Smn′k,j;k−1,2j−1=nmn+m1/2·1n∑i=abIzi≤zk,j−1m∑iL=acIziL≤zk,j=nmn+m1/2W1n∑i=abIzi≤cAk,j−1m∑iL=acIziL≤cAk,j,Sk,j;R=Smn′k,j;k−1,2j=nmn+m1/2·1n∑i=abIzi≤zk,j−1m∑iR=c+1bIziR≤zk,j=nmn+m1/2W1n∑i=abIzi≤cAk,j−1m∑iR=c+1bIziR≤cAk,j,

where 2 ≤ *k* ≤ log_2_ *N*, 1 ≤ *j* ≤ *N*/2^*k*^; *a* = 2^*k*^(*j* − 1) + 1, *b* = 2^*k*^ *j*, *c* = 2^*k*^(*j* − 1) + 2^(*k*−1)^; *n* = 2^*k*^, *m* = 2^*k*−1^; and $W=(\sqrt{2})\overset{\wedge }{\;}k/2^{k}$. To estimate an optimal path towards the potential change position within *Z*, without loss of generality, the first search criterion is introduced based on the statistic fluctuations *S*_*k*,*j*;*L*_ and *S*_*k*,*j*;*R*_.

**Criterion 1 .**

Given two statistic fluctuation variables *S*_*k*,*j*;*L*_ and *S*_*k*,*j*;*R*_ in accordance with two non-leaf child nodes cA_*k*−1,2*j*−1_ and cA_*k*−1,2*j*_ of the current selected node cA_*k*,*j*_ in BSTcA, and 2 ≤ *k* ≤ log_2_ *N*,

(a) if (*S*_*k*,*j*;*L*_ > *S*_*k*,*j*;*R*_)∧(*S*_*k*,*j*;*L*_ > *C*(*α*)) holds true, then the left-child node cA_*k*−1,2*j*−1_ is selected and involved into the current search path; meanwhile, the right-child cA_*k*−1,2*j*_ is discarded;
(b) if (*S*_*k*,*j*;*R*_ > *S*_*k*,*j*;*L*_)∧(*S*_*k*,*j*;*R*_ > *C*(*α*)) holds true, then the right-child node cA_*k*−1,2*j*_ is selected and involved into the current search path; meanwhile, the left-child cA_*k*−1,2*j*−1_ is discarded.

**Proof**

**Figure 3:**
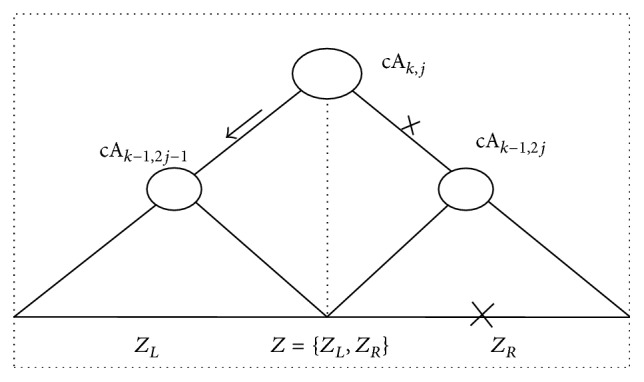
The scheme of Criterion 1 based on the statistic fluctuations within BSTcA. In terms of this criterion, the left or right-child node, that is, cA_*k*,2*j*−1_ or cA_*k*,2*j*_, might be selected to be involved in the current search path; meanwhile the remaining one is discarded. Thereafter, an optimal path towards the potential abrupt change in *Z* is expected to be obtained from BSTcA, after log_2_⁡*N* binary search steps.

For a selected non-leaf node cA_*k*,*j*_ in BSTcA, as shown in Figure 3, the original time series *Z* is divided equally into two adjacent segments *Z*_*L*_ and *Z*_*R*_, which are covered by two non-leaf child nodes cA_*k*−1,2*j*−1_ and cA_*k*−1,2*j*_, respectively. According to the definitions of *S*_*k*,*j*;*L*_ and *S*_*k*,*j*;*R*_ in (10), the satisfied *S*_*k*,*j*;*L*_ > *S*_*k*,*j*;*R*_ indicates that the statistic fluctuation within *Z*_*L*_ is more significant than that one within *Z*_*R*_; that is, a potential abrupt change might be contained in *Z*_*L*_ with more probability than in *Z*_*R*_, and vice versa. Furthermore, if *S*_*k*,*j*;*L*_ > *C*(*α*) holds true, then (*H*_1_) of Hypothesis 1 is satisfied; that is, abrupt change occurs in *Z*_*L*_, and vice versa, where *C*(*α*) is the critical value predefined in an identical distribution and *α* is the significance level. Therefore, one of the two child nodes cA_*k*−1,2*j*−1_ and cA_*k*−1,2*j*_ is selected and involved into the current search path; meanwhile, the remaining one is discarded. Once the statistic fluctuation is significant enough, an optimal search path can be detected by Criterion 1 from the top to the last non-leaf level in BSTcA. However, the search procedure is probably forced to cease because the statistic fluctuation is so insignificant that Criterion 1 is invalid for detecting it, especially for the left or the right boundary when sample *Z* is with smaller size *N*. Therefore, it is necessary to introduce another search criterion based on the variance fluctuations within BSTcD.

**Definition 4 .**

For a current non-leaf node cD_*k*,*j*_ in BSTcD, with its left and right-child nodes cD_*k*−1,2*j*−1_ and cD_*k*−1,2*j*_, respectively, the variance fluctuations *D*_*k*,*j*;*L*_ and *D*_*k*,*j*;*R*_ are defined in terms of (4) as

(11) Dk,j;L=Dmn′k,j;k−1,2j−1=nmn+m1/2·1n∑iL=acziL−∑iR=c+1bziR−1m∑La=alczLa−∑Lb=lc+1czLb=nmn+m1/2N′cDk,j−M′cDk−1,2j−1,Dk,j;R=Dmn′k,j;k−1,2j=nmn+m1/2·1n∑iL=acziL−∑iR=c+1bziR−1m∑Ra=c+1rczRa−∑Rb=rc+1bzRb=nmn+m1/2N′cDk,j−M′cDk−1,2j,

where 2 ≤ *k* ≤ log_2_ *N*, 1 ≤ *j* ≤ *N*/2^*k*^; *a* = 2^*k*^(*j* − 1) + 1, *b* = 2^*k*^ *j*, *c* = 2^*k*^(*j* − 1) + 2^(*k*−1)^; *lc* = 2^*k*^(*j* − 1) + 2^(*k*−2)^, *rc* = *c* + 2^(*k*−2)^; *n* = 2^*k*^, *m* = 2^*k*−1^; and $N^{\prime }=(\sqrt{2})\overset{\wedge }{\;}k/2^{k}$, $M^{\prime }=(\sqrt{2})\overset{\wedge }{\;}(k-1)/2^{\left(k-1\right)}$.

Suppose Criterion 1 is invalid as (*S*_*k*,*j*;*L*_ = *S*_*k*,*j*;*R*_)||(max(*S*_*k*,*j*;*L*_, *S*_*k*,*j*;*R*_) ≤ *C*(*α*)) holds true; the second search criterion needs to be introduced in terms of the two variance fluctuation variables *D*_*k*,*j*;*L*_ and *D*_*k*,*j*;*R*_ as follows.

**Criterion 2 .**

Given two variance fluctuation variables *D*_*k*,*j*;*L*_ and *D*_*k*,*j*;*R*_ according to the two non-leaf child nodes cD_*k*−1,2*j*−1_ and cD_*k*−1,2*j*_ of the selected node cD_*k*,*j*_ in BSTcD, and 2 ≤ *k* ≤ log_2_ *N*,

(a) if (*D*_*k*,*j*;*L*_ > *D*_*k*,*j*;*R*_)∧(*D*_*k*,*j*;*L*_ > *C*(*β*)) holds true, then the left-child node cA_*k*−1,2*j*−1_ in BSTcA is accordingly selected and involved into the current search path; meanwhile the right one is ignored;
(b) if (*D*_*k*,*j*;*L*_ < *D*_*k*,*j*;*R*_)∧(*D*_*k*,*j*;*R*_ > *C*(*β*)) holds true, then the right-child node cA_*k*−1,2*j*_ in BSTcA is accordingly selected and involved into the current search path; meanwhile the left one is ignored.

**Proof**

**Figure 4:**
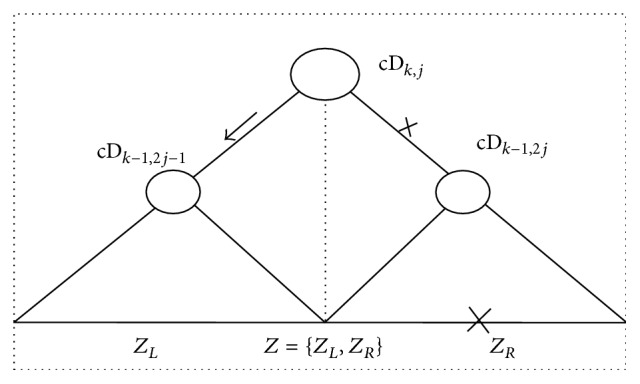
The scheme of Criterion 2 based on the variance fluctuations within BSTcD. Supposing Criterion 1 is invalid for insignificant statistic fluctuation within BSTcA, Criterion 2 ensures that one of the two non-leaf child nodes cA_*k*−1,2*j*−1_ and cA_*k*−1,2*j*_ can also be selected from BSTcA, in accordance with the variance fluctuation within BSTcD. Therefore, the search procedure can keep going forward, to the potential abrupt change in *Z*.

Similarly, as illustrated in Figure 4, the satisfied (*D*_*k*,*j*;*L*_ > *D*_*k*,*j*;*R*_) in Criterion 2 means that the variance fluctuations within *Z*_*L*_ are stronger than that one within *Z*_*R*_, in terms of the definitions of *D*_*k*,*j*;*L*_ and *D*_*k*,*j*;*R*_ in (11). That is, a potential abrupt change might exist in *Z*_*L*_ with more probability than in *Z*_*R*_, and vice versa. Meanwhile, if *D*_*k*,*j*;*L*_ > *C*(*β*) holds true, then (*H*_1_) in Hypothesis 2 is satisfied; that is, abrupt change occurs in *Z*_*L*_, and vice versa, where *C*(*β*) is the critical value predefined in an identical distribution and *β* is the significance level. As a result, one of the two non-leaf child nodes cA_*k*−1,2*j*−1_ and cA_*k*−1,2*j*_ in BSTcA can be accordingly selected, and the other one is neglected. Therefore, if Criterion 1 is invalid for less significant statistic fluctuation within BSTcA, Criterion 2 ensures that the search procedure can keep going forward to the potential abrupt change in *Z*, especially when abrupt change occurs near the left or the right boundary of *Z* with smaller size *N*.

Based on Criterions 1 and 2 above, a search path can be obtained from the top root to the last non-leaf levels of BSTcA. In order to estimate an abrupt change from the original elements of *Z*, another criterion needs to be introduced to discern which one can be selected from two adjacent leaf nodes in BSTcA.

**Definition 5 .**

Supposing the current node cA_*k*,*j*_ is selected in the last non-leaf level of BSTcA, *k* = 1, with two child leaf nodes *z*_2*j*−1_ and *z*_2*j*_, two statistic fluctuation variables *D*_*L*_ and *D*_*R*_ are defined based on KS test as

(12) DL=DmnzL=mnm+n1/2FmzL−GnzL=mnm+n1/2·1m∑i=12j−1Izi≤zL−1n∑h=2jNIzh≤zL,DR=DmnzR=mnm+n1/2FmzR−GnzR=mnm+n1/2·1m∑i=12jIzi≤zR−1n∑h=2j+1NIzh≤zR,

where *z*_*L*_ = *z*_2*j*−1_ and *z*_*R*_ = *z*_2*j*_; *F*_*m*_(*z*) and *G*_*n*_(*z*) refer to the e.c.d.f of *Z*_*L*_ = {*z*_1_,…, *z*_*m*_} and *Z*_*R*_ = {*z*_*m*+1_,…, *z*_*N*_}, respectively; *m* = 2*j* − 1 or 2*j* and *n* = *N* − *m*.

Consider that the largest statistic fluctuation between *F*_*m*_(*x*) and *G*_*n*_(*x*) is achieved either before or after one of the jumps, that is,

(13) supx∈RGnx−Fmx=max1≤i≤nFmzi−−Gnzi−before  the  ith  jumpFmzi−Gnziafter  the  ith  jump.

Then, another two variables *D*_*L*_^−^ and *D*_*R*_^−^ are defined as

(14) DL−=DmnzL−=mnm+n1/2FmzL−−GnzL−=mnm+n1/2·1m∑i=12j−1Izi<zL−1n∑h=2jNIzh<zL,DR−=DmnzR−=mnm+n1/2FmzR−−GnzR−=mnm+n1/2·1m∑i=12j−1Izi<zR−1n∑h=2jNIzh<zR.

Therefore, the maximal statistic fluctuations *D*_*L*_′ and *D*_*R*_′ can be selected from *D*_*L*_^−^ and *D*_*L*_, as well as *D*_*R*_^−^ and *D*_*R*_. Then, the third search criterion is introduced in terms of *D*_*L*_′ and *D*_*R*_′ as follows.

**Criterion 3 .**

Given *D*_*L*_′ and *D*_*R*_′ in accordance with two child leaf nodes *z*_2*j*−1_ and *z*_2*j*_ of the selected non-leaf node cA_*k*,*j*_ in BSTcA, *k* = 1,

(a) if (max(*D*_*L*_′, *D*_*R*_′) = *D*_*L*_′)∧(*D*_*L*_′ > *C*(*γ*)) holds true, then the left leaf node *z*_2*j*−1_ in *Z* is taken as the estimated CP, and the right one is neglected;
(b) if (max(*D*_*L*_′, *D*_*R*_′) = *D*_*R*_′)∧(*D*_*R*_′ > *C*(*γ*)) holds true, then the right leaf node *z*_2*j*_ in *Z* is taken as the estimated CP, and the left one is neglected;
(c) otherwise, no abrupt change is detected from *Z*.

**Proof**

Obviously, if max(*D*_*L*_′, *D*_*R*_′) > *C*(*γ*) is satisfied in Criterion 3, then the statistic fluctuation overtakes the critical value *C*(*γ*) which is given in an identical data distribution, and *γ* is the significance level. Therefore, one of the two leaf nodes *z*_2*j*−1_ and *z*_2*j*_ is taken as the estimated CP within *Z*.

Supposing a non-leaf node cA_*k*,*j*_ is selected in BSTcA, the statistic and variance fluctuations are accordingly calculated between two adjacent segments *Z*_*L*_ and *Z*_*R*_. Meanwhile, the search procedure is implemented from the root to non-leaf nodes in the last second level of BSTcA, in terms of Criterions 1 and 2. Then, the estimated CP can be obtained from the leaf nodes in BSTcA, by using Criterion 3. Thereafter, an optimal path towards a potential CP within *Z* is detected from BSTcA, after about log_2_ *N* binary search steps.

### 3.3. Methods Compared with BSTKS

There are many methods proposed for abrupt change detection in time series, and the following are some typical methods, to evaluate the proposed BSTKS framework.

*KS Statistic* (see [31]). In this method, a diagnosed time series *Z* is divided into two adjacent segments *Z*_*L*_ = {*z*_1_, *z*_2_,…, *z*_*m*_} and *Z*_*R*_ = {*z*_*m*+1_, *z*_*m*+2_,…, *z*_*N*_}, and then KS statistic is applied to calculate the statistic distance between *Z*_*L*_ and *Z*_*R*_ as

(15) Dmnx=mnN1/2supx∈RFnx−Gmx=mnN1/2supx∈R∑R=m+1nIzR<x−∑L=1mIzL<x,

where *F*_*n*_(*x*) and *G*_*m*_(*x*) stand for the e.c.d.f of *Z*_*L*_, and *Z*_*R*_, respectively; *N* = *m* + *n*, *N* is the total length of *Z*, and *m* refers to a current test position within *Z*.

*t-Statistic* (see [32]). *t* also known as Welch's *t*-test is used only when the two population variances are assumed different (the two sample sizes may or may not be equal) and hence must be estimated separately. Suppose a diagnosed *Z* is divided into *Z*_*L*_ = {*z*_1_, *z*_2_,…, *z*_*m*_} and *Z*_*R*_ = {*z*_*m*+1_, *z*_*m*+2_,…, *z*_*N*_}. Then, *t*-statistic is calculated as

(16) $$\begin{array}{c} t=\frac{{\overset{-}{Z}}_{L}-{\overset{-}{Z}}_{R}}{S_{{\overset{-}{Z}}_{L}-{\overset{-}{Z}}_{R}}},\;S_{{\overset{-}{Z}}_{L}-{\overset{-}{Z}}_{R}}=\sqrt{\frac{S_{1}^{2}}{m}+\frac{S_{2}^{2}}{n}}, \end{array}$$

where ${\overset{-}{Z}}_{L}$ and ${\overset{-}{Z}}_{R}$ are the sample means of *Z*_*L*_ and *Z*_*R*_, respectively; *S* is an unbiased estimator of the standard deviation, *N* = *m* + *n*, and *m* and *n* are the sizes of two segments *Z*_*L*_ and *Z*_*R*_, respectively.

*SSA* (see [12, 13, 33]). In SSA method, a windowed portion is chosen within a time series *Z* = {*z*_1_, *z*_2_,…, *z*_*N*_}, where *N* is large enough and a window width *m* and the lag parameter *M* are set such that *M* = *m*/2, *K* = *m* − *M* + 1. For each *n* = 0,1,…, *N* − *m* − *M*, this method takes an interval of the time series [*n* + 1, *n* + *m*] and then defines the *M* × *K* trajectory matrix *Xn* and describes the structure of the windowed portion as an *L*-dimensional subspace. If the structure changes further, it will not be well described by the computed subspace. Then, the distance between this subspace and the new trajectory vectors will increase; therefore, this increase will signal that an abrupt change occurs in *Z*.

## 4. Results and Discussion

In this section, the proposed BSTKS is evaluated on the synthetic time series and real EEG recordings with different size *N*. By comparing with existing KS, *t*, and SSA methods, the efficiency, sensitivity, and performance are analyzed in terms of the computation time, error and accuracy, hit rate, and AUC of ROC analyses. Furthermore, the novelty of our algorithm and necessity for real application are discussed in the following paragraphs.

### 4.1. CP Detection on Synthetic Time Series

In our simulations, some typical time series samples were derived from the normally distributed datasets (mean, *u* = 0, and standard deviation, sd = 1). Each diagnosed sample of size *N* is composed of a normal segment of size *k* and an adjacent segment of size *N* − *k*, in which the abnormal part is simulated by adding a constant variation *v* into the random numbers of size *N* − *k*. The proposed BSTKS and other three methods, namely, KS, *t*, and SSA, were tested, respectively, on 200 samples which were derived from each time series group *G*_*i*_ with $N_{i}=2\overset{\wedge }{\;}(4+i)$, *i* = 1,2,…, 7, and *v*_*i*_ = *d*(1 + log_2_(*k* − 4)), where *k* = log_2_(*N*_*i*_) and *d* = 1.0. For each sample in *G*_*i*_, a series of test positions were arranged by $CPK_{j}=j*(2\overset{\wedge }{\;}(k-4))$, *k* ≥ 5, and *j* = 1,2,…, 15.

**Figure 5:**
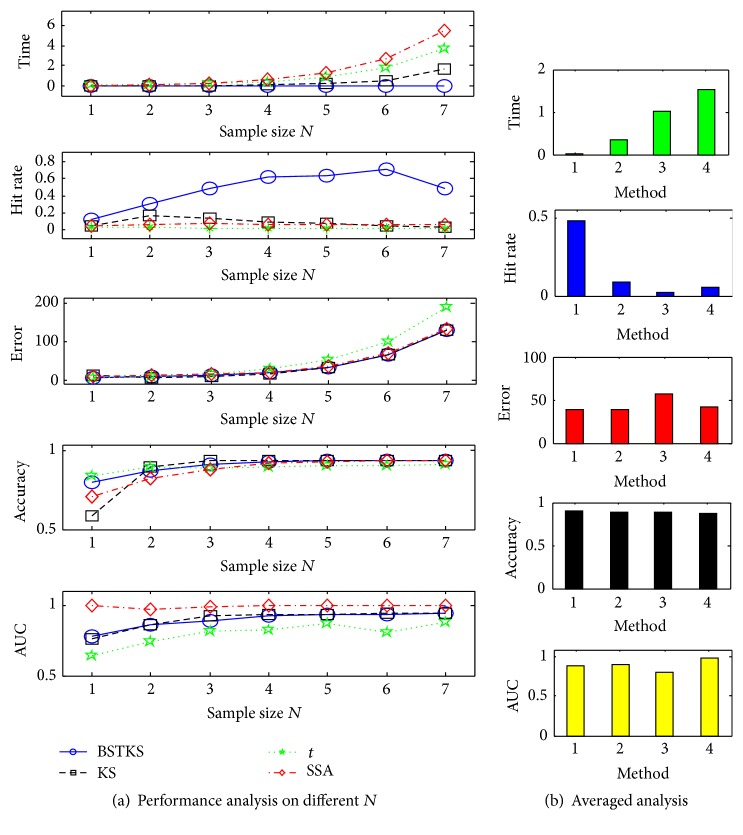
The simulations on *G*_1_ to *G*_7_ with size *N* from 2^5^ to 2^11^. (a) The results in terms of computation time, hit rate, error and accuracy, and AUC of ROC analyses. (b) The average analyses on BSTKS and other three methods. In the histograms, “1,” “2,” “3,” and “4” stand for BSTKS, KS, *t*, and SSA, respectively.

First, simulations were carried out according to different value of sample size *N*_*i*_ and test position *CPK*_*j*_. The average analyses on four methods were listed in Table 1, and the results of simulations on datasets *G*_1_–*G*_7_ were illustrated in Figure 5. In general, our BSTKS is the most promising with the shortest computation time, the highest hit rate, the smallest error, and the highest accuracy out of all four methods. Particularly, as sample size *N* increases from *N*_1_ to *N*_7_, all four methods take longer time for bigger *N*, and BSTKS is always the fastest one. Meanwhile, BSTKS owns the highest level of hit rate against the low tracks of other three methods; and BSTKS is much more efficient with the smallest error and the highest accuracy, though all four methods tend to be better with *N* increasing. However, BSTKS has smaller AUC of ROC analyses, that is, bigger search space, than SSA and KS.

**Figure 6:**
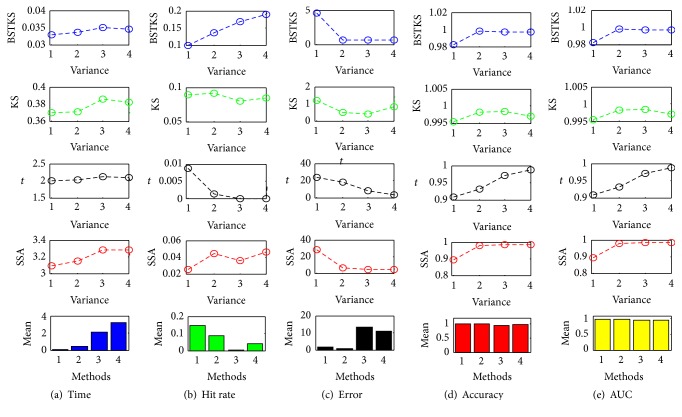
The simulations on 200 samples in *G*_4_ with different variances. Under different variances *v* from 0.5 to 3.0, (a) the computation time, (b) the hit rate, (c) the error, (d) the accuracy, and (e) the AUC of ROC analysis, for BSTKS, KS, *t*, and SSA, respectively. In all “mean” histograms, “1,” “2,” “3,” and “4” in *x*-axis stand for BSTKS, KS, *t*, and SSA methods, respectively.

Second, simulations were carried out based on the datasets *G*_1_, *G*_4_, and *G*_7_. The proposed BSTKS and other three methods were tested according to the different value of variance *v* = *d*(1 + log⁡2(*k* − 4)), *k* = 5,8, 11, and *d* = 0.5,1.0,2.0,3.0, respectively. The average results of four methods on *G*_1_, *G*_4_, and *G*_7_ were summarized in Table 2, and the typical simulations were selected on *G*_4_ and represented in Figure 6. Generally, when *v* gets larger, all four methods get better hit rate, accuracy, and AUC of ROC analysis, except for longer computation time for bigger size *N*. Compared with other three methods, the proposed BSTKS is more encouraging because of the shortest computation time, especially when *N* gets bigger, as well as the highest hit rate and accuracy, especially when *N* gets smaller. Moreover, the simulations on *G*_4_ with different variance *v* (Figure 6) explicitly illustrate that BSTKS has the best performance when *v* gets larger, in terms of the shortest time and the biggest increase of the hit rate out of four methods. For the accuracy and AUC, both BSTKS and KS keep higher sensitivity than *t* and SSA, as *v* increases from 0.5 to 3.0. Moreover, the simulations on *G*_1_ and *G*_7_ were omitted, because similar results can be obtained like *G*_4_ above.

**Figure 7:**
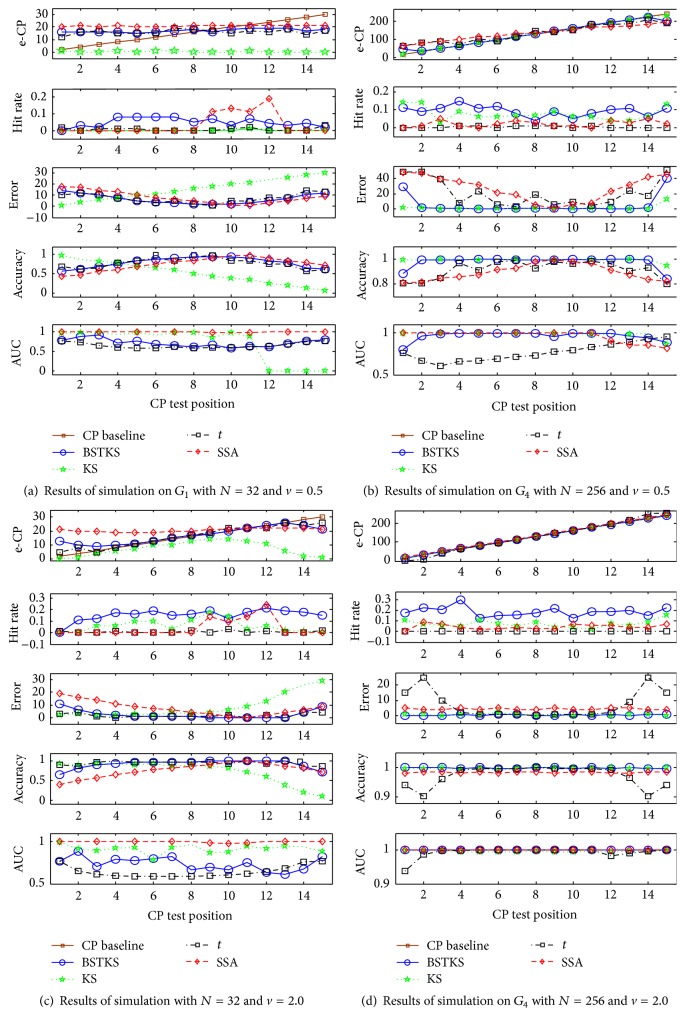
The simulations on *G*_1_ and *G*_4_ according to the different variance *v* and test position* CPK*. The results were shown in (a) *G*_1_ with *N* = 32 and *v* = 0.5, (b) *G*_4_ with *N* = 256 and *v* = 0.5, (c) *G*_1_ with *N* = 32 and *v* = 2.0, and (d) *G*_4_ with *N* = 256 and *v* = 2.0, in terms of e-CP, hit rate, error, accuracy, and AUC, respectively.

**Figure 8:**
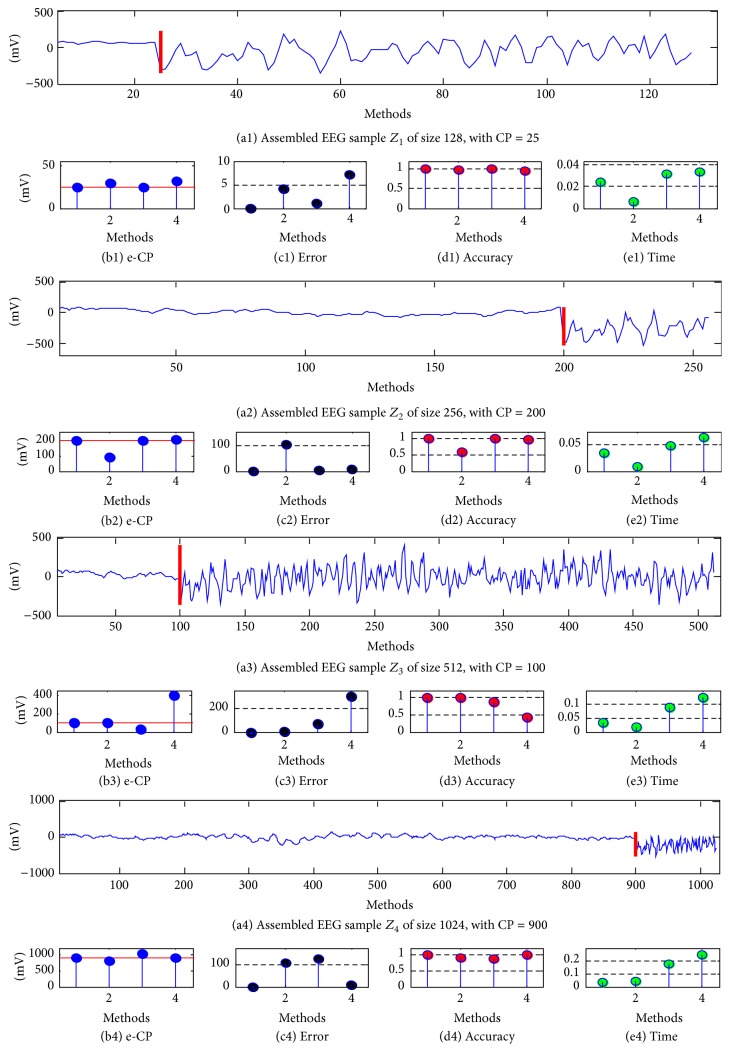
The results of CP detection on the assembled EEG samples *Z*_1_–*Z*_4_ with different value of sample size *N* and test position* CPK*. For *Z*_1_–*Z*_4_ with different *N* from 2^7^ to 2^10^, (a1–a4) the assembled EEG samples *Z*_1_–*Z*_4_ with the assigned test position* CPK*, (b1–b4) the e-CP, (c1–c4) the error of e-CP, (d1–d4) the accuracy of e-CP, and (e1–e4) the computation time. In the *x*-axis of (b–e), the methods “1,” “2,” “3,” and “4” stand for BSTKS, KS, *t*, and SSA, respectively.

Third, simulations were implemented based on different CP test positions within *G*_1_ and *G*_4_. The proposed BSTKS and other three methods were analyzed according to the different value of test position* CPK* and variance *v*. The results of simulations on *G*_1_ and *G*_4_ were illustrated in Figure 7, and the results near the left and right boundaries in *G*_1_ and *G*_4_ were summarized in Table 3. In general, all four methods tend to be better, when *N* increases under a fixed *v*, or when *v* increases under a fixed *N*. Meanwhile, for test position* CPK* near the left and right boundaries, the proposed BSTKS produces better performance than other three methods, because of the highest hit rate, the smallest error in all four methods, and higher accuracy and AUC than *t* and SSA. Moreover, the simulations on *G*_1_ and *G*_4_ near the left and right boundaries were illustrated in Figure 8 in detail. In terms of the distribution of estimated CP (e-CP), PDF of e-CP, and AUC of ROC analysis, these simulations indicate that BSTKS is more sensitive for both left and right boundaries than other three methods, especially when sample size *N* and variance *v* get smaller.

Therefore, all simulation results above suggest that our proposed BSTKS is an encouraging and efficient method for abrupt change detection from the synthetic time series datasets, because of the shortest computation time, the highest hit rate, and accuracy out of four methods, especially for less significant statistic fluctuation when *N* gets smaller, as well as for less significant variance fluctuation when *N* gets bigger, and *v* gets smaller.

### 4.2. Abrupt Change Analyses on EEG Recordings

To verify the proposed method further, we take some representative samples from the CHBMIT Scalp EEG Database. In the PhysioBank platform, the CHBMIT Scalp EEG Database (CHBMIT) was collected at the Children's Hospital Boston; it consists of EEG recordings from pediatric subjects with intractable seizures [34, 35]. In this CHBMIT EEG database, some subjects were monitored up to several days after withdrawal of antiseizure medication in order to characterize their seizures and assess their candidacy for surgical intervention. Based on these EEG recordings in the CHBMIT EEG database, as well as some existing experiments in [36–39], the proposed BSTKS and other three methods were tested according to different value of test position* CPK* and sample size *N*.

First, a diagnosed EEG sample *Z* = [*Z*_*L*_, *Z*_*R*_] was assembled from two significantly different segments, in which *Z*_*L*_ = {*z*_1_,…, *z*_*CPK*_} and *Z*_*R*_ = {*z*_*CPK*+1_,…, *z*_*N*_} were derived from chb01_04_edfm and chb01_05_edfm, respectively. Then, BSTKS and other three methods were tested on the assembled EEG recordings *Z*_1_–*Z*_8_, respectively, according to the different value of assigned test position* CPK* and sample size *N*. The results of abrupt change detection on these assembled EEG samples were illustrated in Figure 8 and summarized in Table 4. Generally speaking, all four methods can roughly estimate the assigned test position from each assembled EEG recording and then divide it into two adjacent segments *Z*_*L*_ and *Z*_*R*_. It is worth stressing that the proposed BSTKS can discern the different EEG segments accurately with the smallest error and the highest accuracy out of four methods. Also, BSTKS is the most efficient and encouraging with the shortest time in all four methods.

Moreover, for* CPK* near the left and right boundaries in *Z*_1_–*Z*_8_, BSTKS has much better sensitivity than other KS, *t*, and SSA methods because of the smallest error and the highest accuracy, especially for less statistic fluctuation when *N* gets smaller, as well as less significant variance fluctuation when *N* gets bigger. Supposing the assembled EEG sample indicates that a sharp transition of one's mental situation occurs before and after a sudden attack or acute stimulation, it is meaningful to estimate the location of the abrupt change and the maximal difference of data distribution exists between two adjacent EEG segments. These experiments above suggest that the proposed BSTKS can successfully detect the change position where a sudden change occurs under a potential mental shock, more quickly and efficiently than KS, *t*, and SSA methods.

**Figure 9:**
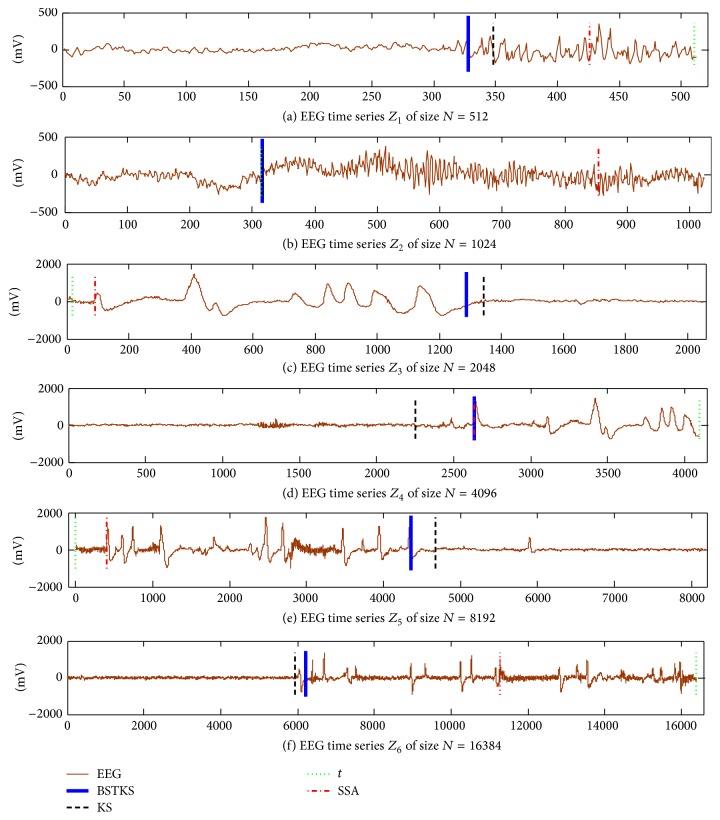
The analyses of abrupt change on the original EEG samples, by BSTKS, KS, *t*, and SSA, respectively. (a–f) The results of CP detection from the original EEG recordings *Z*_1_–*Z*_6_, with *N* from 2^9^ to 2^14^, respectively.

Second, the original EEG samples *Z*_1_–*Z*_6_ were selected directly from different recordings in the chb01_05_edfm; then the proposed BSTKS and other three methods were tested according to different sample size *N*. Because the distance of e.c.d.f (V.e.c.d.f) can partly reflect the data fluctuation between two adjacent EEG segments, we use this V.e.c.d.f variable to distinguish different performance of BSTKS and other three methods. The results of abrupt change analyses on these original EEG recordings were shown in Figure 9 and summarized in Table 5. For all methods above, they can estimate an abrupt change from each of these original EEG samples *Z*_1_–*Z*_6_ and then divide it into two adjacent EEG segments. Compared with other three methods, the proposed BSTKS is encouraging for the shortest time out of four methods. Moreover, BSTKS has bigger V.e.c.d.f than *t* and SSA, which means that it can more reasonably distinguish two adjacent EEG segments with different state of mental health. Although KS has the biggest V.e.c.d.f in all four methods, it takes much more search time than BSTKS, especially when sample size *N* is getting larger. In addition, *t* needs the longest search time out of four methods, and it is invalid occasionally, for example, for *Z*_1_, *Z*_5_, and *Z*_6_.

For these original EEG recordings with intractable seizures, it is of great concern to predict when and where a significant change happens from these EEG signals. This abrupt change probably indicates that a patient encounters a vertical transition from a previous mental status, and it is very important and helpful for diagnosing the patients with intractable seizures. These experiments on original EEG samples above indicate that the proposed BSTKS can not only accurately detect the change position, but also estimate the maximal difference of data distribution existing between two adjacent EEG segments, more quickly and efficiently than existing KS, *t*, and SSA methods.

## 5. Conclusion

In this paper, a novel BSTKS method is proposed based on binary search trees and a modified KS statistic. In this method, two BSTs were constructed from a diagnosed time series by multilevel HWT, and then an optimal search path is detected from the root to leaf nodes of two BSTs in terms of three search criteria. The novelty of the proposed method is addressed by comparing with other KS, *t*, and SSA methods, and simulations on the synthetic time series indicate that the proposed BSTKS is more efficient due to the shortest time, the highest hit rate, and the smallest error and highest accuracy out of four methods. Meanwhile, BSTKS has better sensitivity than KS near the left and right boundaries, because of shorter search time, higher hit rate, and bigger AUC, especially when sample size *N* gets smaller with less significant statistic fluctuation. In addition, the necessity of the proposed method in the real domain is analyzed on real EEG recordings, and the results indicate that the proposed method can successfully discern an abrupt change and then obviously distinguish two adjacent EEG segments from the real EEG recordings. Through inspecting the significant fluctuation between adjacent segments signals, it is encouraging further for useful information inspection on all kinds of physiological and psychological time series signals. In a word, our BSTKS is a novel, efficient, and promising method for abrupt change analysis, and it is very helpful for useful information inspection on all kinds of real time series with different scales.

## Figures and Tables

**Table 1:** The averaged results on four methods with datasets *G*_1_ to *G*_7_.

	Time	Hit rate	Error	Accuracy	AUC
*BSTKS*	*.0063*	*.4797*	*38.5268*	*.9018*	*.8922*
KS	.3537	.0841	38.7321	.8804	.8984
*t*	1.0068	.0168	56.3036	.8878	.7960
SSA	1.5218	.0583	41.9464	.8762	.9941

**Table 2:** The summary of simulations according to different variances in *G*_1_, *G*_4_, and *G*_7_.

Items		Methods											
		*N* = 2^5^				*N* = 2^8^				*N* = 2^11^			
		***BSTKS***	KS	*t*	SSA	***BSTKS***	KS	*t*	SSA	***BSTKS***	KS	*t*	SSA
*d* = 0.5	Time	***.018***	*.035*	*.227*	*.116*	***.029***	*.335*	*1.85*	*2.81*	***.060***	6.96	17.2	24.6
	Hit rate	***.046***	.005	.010	.038	***.093***	.093	.005	.025	***.106***	.056	.006	.034
	Accuracy	***.792***	.515	.792	.748	***.984***	.995	.905	.899	***.999***	.999	.944	.884
	AUC	***.694***	.694	.644	.997	***.954***	.951	.978	.971	***.983***	1.00	.999	1.00

*d* = 1.0	Time	***.018***	.034	.223	.113	***.031***	.356	1.97	2.98	***.061***	7.09	18.1	26.5
	Hit rate	***.086***	.013	.007	.035	***.041***	.001	.099	.142	***.135***	.045	.000	.035
	Accuracy	***.846***	.552	.839	.756	***.998***	.998	.939	.974	***.999***	.999	.940	.986
	AUC	***.695***	.695	.851	.997	***.993***	.992	.997	.991	***.992***	1.00	.998	1.00

*d* = 2.0	Time	***.018***	.035	.229	.115	***.031***	.345	1.91	2.88	***.065***	7.60	19.7	29.1
	Hit rate	***.165***	.061	.007	.049	***.181***	.090	.000	.049	***.167***	.028	.000	.053
	Accuracy	***.927***	.737	.958	.765	***.998***	.997	.971	.983	***.999***	.999	.984	.997
	AUC	***.754***	.754	.908	.997	***.998***	.999	.996	1.00	***.995***	1.00	.999	1.00

*d* = 3.0	Time	***.019***	.037	.245	.125	***.034***	.382	2.08	3.28	***.067***	8.08	20.5	31.4
	Hit rate	***.225***	.086	.002	.037	***.189***	.084	0.00	.046	***.169***	.035	0.00	.045
	Accuracy	***.942***	.818	.952	.773	***.997***	.996	.986	.983	***.999***	.999	.991	.998
	AUC	***.857***	.938	.655	.997	***1.00***	.996	.728	.100	***1.00***	.999	.467	1.00

**Table 3:** The summary of simulations on *G*_1_ and *G*_4_ near the left and right boundaries according to different variance *d*.

Items		Methods															
		*N* = 2^5^, *CPK* = 8				*N* = 2^5^, *CPK* = 24				*N* = 2^8^, *CPK* = 16				*N* = 2^8^, *CPK* = 240			
		***BSTKS***	KS	*t*	SSA	***BSTKS***	KS	*t*	SSA	***BSTKS***	KS	*t*	SSA	***BSTKS***	KS	*t*	SSA
*d* = 0.5	Hit rate	***.200***	*.055*	*.036*	*.160*	***.215***	*.060*	*.010*	*0.0*	***.230***	.160	0.0	0.0	***.265***	.165	0.0	.065
	Error	***2***	20	8	3	***3***	7	8	12	***24***	1	134	49	***28***	21	102	33
	Accuracy	***.937***	.375	.750	.906	***.906***	.781	.750	.625	***.960***	.996	.476	.808	***.891***	.918	.601	.871
	AUC	***.635***	.963	.641	.978	***.656***	.987	.599	.978	***.599***	.946	.780	.797	***.750***	.884	.564	.797

*d* = 1.0	Hit rate	***.515***	.160	.040	.295	***.485***	.190	.005	0.0	***.490***	.195	0.0	0.0	***.535***	.175	.025	.100
	Error	***0***	10	4	2	***0***	3	8	12	***1***	0	112	10	***0***	1	90	1
	Accuracy	***1.0***	.687	.875	.937	***1.0***	.906	.750	.625	***.996***	1.0	.562	.960	***1.00***	.996	.648	.996
	AUC	***.831***	.883	.641	.978	***.922***	.927	.599	.978	***.864***	.986	.780	.829	***.999***	.988	.657	.911

*d* = 2.0	Hit rate	***.655***	.240	0.0	.220	***.725***	.175	0.0	0.0	***.510***	.160	0.0	0.0	***.530***	.150	.005	.065
	Error	***0***	6	1	2	***0***	1	4	12	***0***	0	107	5	***0***	1	36	4
	Accuracy	***1.00***	.812	.968	.937	***1.0***	.968	.875	.625	***1.0***	1.0	.582	.980	***1.0***	.996	.859	.984
	AUC	***.976***	.979	.770	.978	***.938***	.875	.808	.978	***.978***	.985	.780	.999	***1.0***	.990	.752	.995

*d* = 3.0	Hit rate	***.715***	210	0.0	.265	***.730***	.215	0.0	0.0	***.530***	.195	0.0	0.0	***.545***	.145	0.0	.060
	Error	***0***	6	1	2	***0***	1	1	13	***0***	0	119	5	***0***	2	11	4
	Accuracy	***1.0***	.812	.968	.937	***1.0***	.968	.968	.593	***1.0***	1.0	.535	.980	***1.0***	.992	.957	.984
	AUC	***.999***	.960	.770	.978	***.996***	.822	.808	.978	***.999***	.940	.780	.999	***1.0***	.990	.752	.998

**Table 4:** The summary of abrupt change detection on *Z*_1_–*Z*_8_.

M	*Z*									Mean
	*N*	2^7^	2^8^	2^9^	2^10^	2^7^	2^8^	2^9^	2^10^	
	*CPK*	25	50	100	200	100	200	400	900	
e-CP	***BSTKS***	***25***	***50***	***100***	***276***	***100***	***200***	***376***	***900***	***NA***
	KS	29	36	95	206	34	92	301	795	NA
	*t*	24	255	31	1023	31	199	33	1023	NA
	SSA	32	55	398	1007	106	208	500	907	NA

Err	***BSTKS***	***0***	***0***	***0***	***76***	***0***	***0***	***24***	***0***	***12.5***
	KS	4	14	5	6	66	108	99	105	50.9
	*t*	1	205	69	823	69	1	367	123	207.3
	SSA	7	5	298	807	6	8	100	7	154.8

Acc	***BSTKS***	***1.0***	***1.0***	***1.0***	***.93***	***1.0***	***1.0***	***.95***	***1.0***	***.98***
	KS	.97	.94	.99	.99	.48	.57	.81	.89	.83
	*t*	.99	.20	.86	.20	.46	.99	.28	.88	.61
	SSA	.94	.97	.42	.21	.94	.97	.80	.99	.78

Time	***BSTKS***	***.023***	***.031***	***0.034***	***.036***	***.028***	***.033***	***.035***	***.038***	***.032***
	KS	.019	.021	.038	.049	.020	.029	.039	.052	*.033*
	*t*	.03	.063	.088	.170	.031	.050	.081	.174	*.086*
	SSA	.037	.071	.126	.239	.035	.065	.118	.245	*.117*

**Table 5:** The summary of CP detection from the original EEG samples *Z*_1_–*Z*_6_.

M		*N*						Mean
		2^9^	2^10^	2^11^	2^12^	2^13^	2^14^	
e-CP	***BSTKS***	***328***	***316***	***1286***	***2633***	***4352***	***6224***	***NA***
	KS	348	317	1342	2252	4673	5947	*NA*
	*t*	511	314	17	4095	10	16383	*NA*
	SSA	426	854	90	2634	408	11271	*NA*

V.e.c.d.f	***BSTKS***	***.0649***	***.2608***	***.2822***	***.0997***	***.1318***	***.0388***	***.1464***
	KS	.4603	.3829	.4407	.3050	.3325	.2234	*.3574*
	*t*	0	.1257	.5384	0	0	0	*.1106*
	SSA	.1368	.0850	.1260	.0745	.0212	.0012	*.0741*

Time	***BSTKS***	***.020***	***.020***	***.024***	***.030***	***.019***	***.0320***	***.0241***
	KS	.016	.041	.112	.466	1.461	5.638	*1.289*
	*t*	.072	.137	.281	.913	1.726	4.709	*1.306*
	SSA	.107	.209	.415	1.103	1.769	3.548	*1.192*

## References

[1] Bolton R. J., Hand D. J. (2002). Statistical fraud detection: a review. *Statistical Science*.

[2] Brodsky B. E., Darkhovsky B. S. (1993). *Nonparametric Methods in Change-point Problems*.

[3] Brodsky B. E., Darkhovsky B. S. (2000). *Non-Parametric Statistical Diagnosis: Problems and Methods*.

[4] Yamanishi K., Takeuchi J.-I., Williams G., Milne P. (2004). On-line unsupervised outlier detection using finite mixtures with discounting learning algorithms. *Data Mining and Knowledge Discovery*.

[5] Murad U., Pinkas G. (1999). Unsupervised profiling for identifying superimposed fraud. *Principles of Data Mining and Knowledge Discovery*.

[6] Reeves J., Chen J., Wang X. L., Lund R., Lu Q. (2007). A review and comparison of changepoint detection techniques for climate data. *Journal of Applied Meteorology and Climatology*.

[7] Wang Y., Wu C., Ji Z., Wang B., Liang Y. (2011). Non-parametric change-point method for differential gene expression detection. *PLoS ONE*.

[8] Basseville M. E., Nikiforov I. V. (1993). *Detection of Abrupt Changes: Theory and Application*.

[9] Qi J.-P., Zhang Q., Zhu Y., Qi J. (2014). A novel method for fast change-point detection on simulated time series and electrocardiogram data. *PLoS ONE*.

[10] Yiou P., Baert E., Loutre M.-F. (1996). Spectral analysis of climate data. *Surveys in Geophysics*.

[11] Vautard R., Ghil M. (1989). Singular spectrum analysis in nonlinear dynamics, with applications to paleoclimatic time series. *Physica D: Nonlinear Phenomena*.

[12] Vaisman L., Zariffa J., Popovic M. R. (2010). Application of singular spectrum-based change-point analysis to EMG-onset detection. *Journal of Electromyography and Kinesiology*.

[13] Moskvina V., Zhigljavsky A. (2003). An algorithm based on singular spectrum analysis for change-point detection. *Communications in Statistics—Simulation and Computation*.

[14] Gustafsson F. (2000). *Adaptive Filtering and Change Detection*.

[15] Alarcon-Aquino V., Barria J. A. (2001). Anomaly detection in communication networks using wavelets. *IEE Proceedings: Communications*.

[16] Fremdt S. (2015). Page's sequential procedure for change-point detection in time series regression. *Statistics*.

[17] Priyadarshana M., Polushina T., Sofronov G. (2015). Hybrid algorithms for multiple change-point detection in biological sequences. *Advances in Experimental Medicine and Biology*.

[18] Nosek K., Szkutnik Z. (2014). Change-point detection in a shape-restricted regression model. *Statistics*.

[19] Vautard R., Yiou P., Ghil M. (1992). Singular-spectrum analysis: a toolkit for short, noisy chaotic signals. *Physica D: Nonlinear Phenomena*.

[20] Khalil M., Duchêne J. (1999). Detection and classification of multiple events in piecewise stationary signals: comparison between autoregressive and multiscale approaches. *Signal Processing*.

[21] Kobayashi M. (2001). Wavelets and their applications in industry. *Nonlinear Analysis: Theory, Methods & Applications*.

[22] Percival D. B., Walden A. T. (2006). *Wavelet Methods for Time Series Analysis*.

[23] Salam M., Mohamad D. (2008). Segmentation of Malay Syllables in connected digit speech using statistical approach. *International Journal of Computer Science and Security*.

[24] Qi J., Ding Y., Zhu Y., Wu Y. (2011). Kinetic theory approach to modeling of cellular repair mechanisms under genome stress. *PLoS ONE*.

[25] Tseng V. S., Chen C.-H., Chen C.-H., Hong T.-P. Segmentation of time series by the clustering and genetic algorithms.

[26] Alarcon-Aquino V., Barria J. (2009). Change detection in time series using the maximal overlap discrete wavelet transform. *Latin American Applied Research*.

[27] Chen S., Liu C. (2015). Eye detection using discriminatory Haar features and a new efficient SVM. *Image and Vision Computing*.

[28] Darkhovski B. S. (1994). *Nonparametric Methods in Change-Point Problems: A General Approach and some Concrete Algorithms*.

[29] Walker J. S. (2002). *A Primer on Wavelets and Their Scientific Applications*.

[30] Fryzlewicz P., Rao S. S. (2014). Multiple-change-point detection for auto-regressive conditional heteroscedastic processes. *Journal of the Royal Statistical Society, Series B: Statistical Methodology*.

[31] Simard R., L'Ecuyer P. (2011). Computing the two-sided Kolmogorov-Smirnov distribution. *Journal of Statistical Software*.

[32] Sørlie T., Tibshirani R., Parker J. (2003). Repeated observation of breast tumor subtypes in independent gene expression data sets. *Proceedings of the National Academy of Sciences of the United States of America*.

[33] Hassani H., Zhigljavsky A. (2009). Singular spectrum analysis: methodology and application to economics data. *Journal of Systems Science and Complexity*.

[34] Goldberger A. L., Amaral L. A., Glass L. (2000). PhysioBank, PhysioToolkit, and PhysioNet: components of a new research resource for complex physiologic signals. *Circulation*.

[35] Shoeb A. H. (2009). *Application of Machine Learning to Epileptic Seizure Onset Detection and Treatment*.

[36] Xia Z. H., Wang X. H., Sun X. M., Wang Q. (2016). A secure and dynamic multi-keyword ranked search scheme over encrypted cloud data. *IEEE Transactions on Parallel and Distributed Systems*.

[37] Xie S. D., Wang Y. X. (2014). Construction of tree network with limited delivery latency in homogeneous wireless sensor networks. *Wireless Personal Communications*.

[38] Li J., Li X., Yang B., Sun X. (2015). Segmentation-based image copy-move forgery detection scheme. *IEEE Transactions on Information Forensics and Security*.

[39] Gu B., Sheng V. S., Tay K. Y., Romano W., Li S. (2015). Incremental support vector learning for ordinal regression. *IEEE Transactions on Neural Networks and Learning Systems*.

